# Acute neurological complications in adult patients with cardiogenic shock on veno-arterial extracorporeal membrane oxygenation support

**DOI:** 10.1186/s43044-020-00053-5

**Published:** 2020-05-24

**Authors:** Mohamed Laimoud, Walid Ahmed

**Affiliations:** 1 King Faisal Specialist Hospital & Research Center, Riyadh, Saudi Arabia; 2 Critical Care Medicine Department, Cairo University, Cairo, Egypt

**Keywords:** VA-ECMO, ICH, intracerebral haemorrhage, Stroke, SOFA, Cardiogenic shock, AKI, acute kidney injury, Lactate

## Abstract

**Background:**

Extracorporeal life support has markedly progressed over the recent years to support patients with severe cardiac and pulmonary dysfunction refractory to conventional management. Many patients developed acute neurological complications while being supported with extracorporeal membrane oxygenation (ECMO). Our objectives were to study the frequencies and outcomes of CNS complications in adult patients with cardiogenic shock on veno-arterial extracorporeal membrane oxygenation (VA-ECMO) and to study the risk factors of these CNS complications. We conducted a retrospective study including adult patients admitted to the cardiac critical care unit with cardiopulmonary instability and supported with VA-ECMO from January 2016 until December 2018 in a tertiary care hospital.

**Results:**

After reviewing 231 patients with ECMO, 67 patients with cardiogenic shock supported with VA-ECMO were included. About 65.7% of the studied patients were supported after cardiothoracic surgeries. About 56.7% of the patients developed acute CNS events. According to brain CT imaging, ischaemic stroke was diagnosed in 14.9% and intracerebral haemorrhage (ICH) was diagnosed in 11.9% of patients while 16.4% of patients with CNS events had negative brain CT imaging. The SOFA score was significantly higher in the group with CNS events at ICU admission and after 48 hours . As compared to patients with ischaemic strokes, patients with ICH were younger with lesser BMI, had higher SOFA scores at admission and at 48 hours of ICU admission, had longer cardiopulmonary bypass and aortic cross clamping times and had more support with central than peripheral VA-ECMO. AF was more frequent in the group with CNS events especially in the ischaemic stroke subgroup. Presence of intracardiac thrombi was more frequent in the ischaemic stroke subgroup. There was no statistically significant difference between both groups regarding ECMO circuit thrombi. The use of IABP and presence of DM were more frequent in the ischaemic stroke subgroup. Patients with neurological events had hypoalbuminaemia and higher blood glucose and serum creatinine levels compared to those without CNS events. The peak lactate level and lactate after 24 hours of ECMO support were significantly higher in those with CNS events. Patients with ICH had significant thrombocytopenia and higher INR with more prolonged aPTT and PTT ratio than those with ischaemic stroke. Patients with neurological events had significant hospital mortality, more mechanical ventilation days and tracheostomy, AKI and haemodialysis compared to those without CNS events, but there were no significant differences between both groups regarding ECMO duration, ICU or post ICU stays nor 1 year mortality.

**Conclusion:**

Acute neurological events are frequent in patients supported with VA-ECMO and associated with significant morbidity and hospital mortality. As compared to ischaemic stroke, ICH is more frequent in younger patients with lesser BMI, central VA-ECMO after cardiothoracic surgeries, thrombocytopenia, and coagulopathy. Our findings may have major implications for the care of patients requiring VA-ECMO.

## Background

Extracorporeal membrane oxygenation is frequently used as an emergency support for patients with cardiopulmonary resuscitation when all other treatment options have failed and mortality is expected. Veno-arterial ECMO involves venous drainage from the femoral vein or right atrium with artificial extracirculatory oxygen exchange. Return to the body is through arterial system via the femoral artery (peripheral ECMO) or ascending aorta (central ECMO) [1, 2].

VA-ECMO is associated with significant morbidity and mortality, and among the complications occurring in VA-ECMO treated patients, brain injury is the most frequent, affecting 8–50% [3–7].

We undertook this retrospective observational study to study the frequencies of acute CNS complications in adult patients with cardiogenic shock on VA-ECMO and to study the outcomes and risk factors of these CNS complications.

## Methods

After getting approval from our Institute Ethics Committee for a waiver of informed consent, we conducted a retrospective observational study using the hospital Integrated Compliance Information System (ICIS). The study included patients from January 2016 to December  2018 who were admitted to the adult cardiac critical care unit with VA-ECMO. Our hospital used the Cardiohelp and Rotaflow ECMO machines with biocompatible Bioline coating circuits. Demographic characteristics of patients and clinical and laboratory data were collected. SOFA score was calculated for all patients at ICU admission and after 48 hours for the assessment of organ failure.

### Anticoagulation management during VA-ECMO

Precannulation checking of complete blood count (CBC) and coagulation profile was routinely done for all patients. The coagulation profile included international normalized ratio (INR), activated partial thromboplastin time (aPTT), antithrombin III (AT III), activated clotting time (ACT), and fibrinogen level.

After a heparin bolus (intravenous 80 units/kg) at ECMO initiation, all patients were continuously infused with unfractionated heparin. If ACT was more than 300 s, there was no need to give heparin bolus, serial measurements of ACT every hour were done until the ACT is below 300 s and then heparin infusion was started.

The heparin dose was adjusted, at least twice daily, according to the aPTT (targeting 1.5–2-fold the normal control value), heparin assay (target 0.3–0.7 units/ml), AT III (goal 80–120%) and clinical tolerance. Platelet transfusions were used to keep count more than 50 (10^9^/L) and cryoprecipitate transfusions were used to keep fibrinogen more than 1 (gm/L).

Heparin was stopped when bleeding occurred and restarted once it was controlled. Bleeding requiring heparin withdrawal was defined as any clinical bleeding (at the ECMO implantation site, central or arterial lines, chest tube output, haemoptysis or gastrointestinal bleeding) with or without haemodynamic instability or haemoglobin decrease, judged meaningfully by the patient’s treating physicians.

The ECMO oxygenator membrane and circuits were checked daily by experienced perfusionists and changed when fibrin deposition or thrombi had deleterious effects on blood oxygenation or intravascular haemolysis without other causes of mechanical haemolysis.

### Control of blood glucose during VA-ECMO support

All diabetic and nondiabetic patients supported with VA-ECMO and admitted to ICU were kept to a tight blood glucose control within a range of 4 to 10 mmol/L in accordance with our hospital protocol and the Society of Thoracic Surgeons Practice Guideline series, Blood Glucose Management During Adult Cardiac Surgery [8]. Insulin infusion was used to achieve blood glucose control and blood glucose monitoring every 1–4 h for at least the first 48 hours of ICU admission.

### Neurological assessment of VA-ECMO-supported patients

According to our hospital protocol, all VA-ECMO-treated patients underwent daily routine neurological assessment by intensivists and nurses at least once daily after withdrawal of sedation including Glasgow Coma Scale assessment, brain stem reflexes, eye opening and pupil sizes and reactivity to light assessed every 4 hours. Also, if any unexpected events like seizures, no awakening or unilateral weakness after sedation withdrawal, a cerebral computed tomography (CT) scan was obtained within a few hours.

Near-infrared spectroscopy (NIRS) is a non-invasive modality routinely applied to VA-ECMO-supported patients in our hospital to obtain a continuous measurement of cerebral oxygenation saturation (rSO2%) by placing a frontal scalp electrode. It detects the concentrations of oxygenated and deoxygenated haemoglobin in the cerebral circulation [9, 10].

Electroencephalogram (EEG) monitoring was used to detect brain wave activity by placing electrodes on the scalp. According to our hospital protocol, EEG is used in case of seizures or delayed awakening with negative CT imaging.

All studied patients were divided according to presence or absence of neurological complications into 2 groups, and then those who developed brain injury according to CT brain were subdivided into ischaemic and haemorrhagic subgroups.

### Statistical analysis

Statistical tests were done using the Statistical Package of Social Science Software program, version 23 (SPSS). Data were summarized using mean and standard deviation for quantitative variables and frequency and percentage for qualitative ones. The normality of data was checked using the Kolmogorov-Smirnov normality test. Comparisons between numerical data were done using Student’s *t* test or Mann-Whitney accordingly. Ordinal data was compared using chi-squared test. *p* values less than 0.05 were considered statistically significant. Graphs were used to illustrate some information. Assessment of the areas under the receiver operating characteristics (ROC) curves was performed.

## Results

### Demographic and clinical data

During the study period, 67 patients with cardiogenic shock supported with VA-ECMO were included. About 65.7% of studied patients had postcardiotomy shock and VA-ECMO support. Central VA-ECMO was used to support 52.2% of patients while peripheral VA-ECMO was used in 47.8% of patients via bi-femoral approach. About 56.7% of the patients developed acute neurological manifestations including deterioration of conscious level, unilateral weakness and convulsions. According to brain CT imaging, ischaemic stroke was diagnosed in 14.9% and intracerebral haemorrhage (ICH) was diagnosed in 11.9% of patients while 16.4% of patients with acute neurological events had negative brain CT imaging (Tables 1 and 2).

**Table 1:** Neurological events of VA-ECMO patients

Studied criteria	All patients; *N* (%)
Neurological events	38 (56.7)
Disturbed conscious level	36 (53.7)
Unilateral weakness	5 (7.46)
Convulsions	12 (17.9)
Intracerebral haemorrhage	8 (11.9)
Ischaemic stroke	10 (14.9)
Negative CT brain	11 (16.4)

**Table 2:** Demographic data of patients on VA-ECMO

Studied parameters		All patients (67), *N* (%)	CNS events (38), *N* (%)	No CNS events (29), *N* (%)	*p* value	Ischaemic stroke (*n* = 10), *N* (%)	ICH (*n* = 8), *N* (%)	*p* value
Age (years)		40.13 ± 14.2	40.84 ± 15.9	39.21 ± 11.67	0.032	44.9 ± 15.1	32.25 ± 11.8	0.01
Sex	Males	49 (73.2)	28 (73.7)	21 (72.4)	0.56	5 (50)	5 (62.5)	0.32
	Females	18 (26.8)	10 (26.3)	8 (27.6)		5 (50)	3 (37.5)	
BMI		25.97 ± 7.25	25 ± 7.23	27.24 ± 7.21	0.65	28.30 ± 7.2	21.38 ± 4.8	0.03
Diabetes mellitus		12 (17.9)	7 (18.4)	5 (17.2)	0.83	4 (40)	0	0.02
Systemic hypertension		19 (28.4)	12 (31.6)	7 (24.1)	0.59	4 (40)	1 (12.5)	0.07
Chronic kidney disease		14 (20.9)	11 (28.)	3 (10.3)	0.08	3 (30)	2 (25)	0.52
History of stroke		3 (4.47)	3 (7.9)	0	0.17	0	2 (25)	0.16
History of epilepsy		1 (1.49)	1 (2.63)	0	0.29	0	1 (12.5)	0.24
Left ventricle EF		33.63 ± 13.84 %	33.76 ± 13.29	33.45 ± 14.76	0.17	37.40 ± 13.8	36.75 ± 16.1	0.19
Pre-ECMO AF		25 (37.3)	15 (39.5)	10 (34.5)	0.8	2 (20)	5 (62.5)	0.04
History of oral anticoagulants		27 (40.3%)	15 (39.5)	12 (41.4)	0.56	3 (30)	4 (50)	0.07
Cardiothoracic surgery		44 (65.7%)	28 (73.6)	16 (55.2)	0.12	6 (60)	7 (87.5)	0.04
Cardiopulmonary bypass time (minutes)		229.9 ± 97.1	245.1 ± 105.6	203.1 ± 75.75	0.16	180.40 ± 117.13	250.5 ± 89.7	0.01
Aortic clamping time (minutes)		149.53 ± 56.46	154.75 ± 58.57	139.08 ± 52.84	0.42	129.00 ± 80.72	173.4 ± 53.6	0.03
IABP		13 (19.4)	9 (23.7)	4 (15.4)	0.24	4 (40)	1 (12.5)	0.03
SOFA score at admission		13.10 ± 3.52	14.34 ± 2.95	11.48 ± 3.59	0.001	13.00 ± 3.49	15.25 ± 1.8	0.001
SOFA score after 48 hours		14.61 ± 5.82	16.82 ± 4.93	11.72 ± 5.73	0.001	15.20 ± 5.69	18.25 ± 4.1	0.001
Type of ECMO	Central	35 (52.2)	22 (57.9)	13 (44.8)	0.33	5 (50)	6 (75)	0.04
	Peripheral	32 (47.8)	16 (42.1)	16 (55.2)		5 (50)	2 (25)	
Days before event		–	10.21 ± 5.7	–	–	9.40 ± 5.19	11.13 ± 7.1	0.21
AF during ECMO		36 (53.7)	26 (68.4)	10 (34.5)	0.007	8 (80)	5 (62.5)	0.03
Intracardiac thrombi		6 (8.9)	5 (13.2)	1 (3.4)	0.2	4 (40)	0	0.04
ECMO circuit thrombi		4 (5.9)	3 (7.9)	1 (3.4)	0.4	1 (10)	0	0.6

The patients with CNS manifestations were statistically older than other groups while there were no significant differences regarding other demographic data. The SOFA score was significantly higher in the group with CNS events at ICU admission and after 48 hours. As compared to patients with ischaemic strokes, patients with ICH were younger with lesser BMI, had higher SOFA scores at admission and at 48 hours of ICU admission, had longer cardiopulmonary bypass and aortic cross-clamping times and had more support with central than peripheral VA-ECMO.

AF was more frequent in the group with CNS events especially in the ischaemic stroke subgroup. The presence of intra-cardiac thrombi was more frequent in the ischaemic stroke subgroup. There was no statistical significant difference between groups regarding ECMO circuit thrombi. The use of IABP and presence of DM were more frequent in the ischaemic stroke subgroup. There was no significant difference between ischaemic and ICH groups regarding the duration of ECMO support before the neurological manifestations (Table 2).

### Laboratory data

#### Laboratory data at initiation of VA-ECMO

Patients with CNS events had more prolonged aPTT and PTT ratio compared with those without CNS events and without other significant laboratory differences. With subgroup analysis, the patients with ICH had significant thrombocytopenia and hyperbilirubinaemia compared to those with ischaemic strokes (Table 3).

**Table 3:** Laboratory criteria at VA-ECMO initiation

Laboratory data	All patients	CNS events	No CNS events	*p* value	Ischaemic stroke	ICH	*p* value
Platelet count (10^9^/L)	149.58 ± 91.56	139.61 ± 88.34	162.66 ± 95.58	0.29	181.7 ± 119.28	97.2 ± 50.2	0.01
aPTT (seconds)	56.6 ± 31.1	64.08 ± 37.31	46.83 ± 16.29	0.01	62.90 ± 43.62	61.63 ± 36.8	0.52
PTT ratio	1.57 ± 0.89	1.79 ± 1.04	1.28 ± 0.53	0.01	1.90 ± 1.2	1.63 ± 1.1	0.36
INR	1.73 ± 0.66	1.68 ± 0.57	1.79 ± 0.77	0.51	1.5 ± 0.53	1.75 ± 0.46	0.38
Fibrinogen level (g/L)	3.10 ± 1.42	2.82 ± 1.38	3.43 ± 1.42	0.09	3 ± 1.3	2.50 ± 1.4	0.07
Base excess	− 9.01 ± 3.9	− 9.68 ± 4.23	− 8.14 ± 3.34	0.09	− 10.20 ± 3.25	− 10.38 ± 4.6	0.18
Serum lactate (mmol/L)	6.27 ± 2.22	6.68 ± 2.18	5.72 ± 2.61	0.08	6.60 ± 1.9	6.50 ± 2.5	0.39
Serum bilirubin (μmol/L)	44.73 ± 34.26	44.82 ± 47.84	44.62 ± 41.02	0.51	21.4 ± 8.2	82.50 ± 81.2	0.04
Serum creatinine (μmol/L)	116.55 ± 75.25	117.26 ± 82.77	115.62 ± 65.50	0.91	124.23 ± 74.2	99.5 ± 69.7	0.27
Blood urea (mmol/L)	10.2 ± 6.63	10.03 ± 6.67	10.41 ± 6.38	0.81	8.80 ± 6.8	10.63 ± 8.8	0.09
Serum albumin (g/L)	31.9 ± 6.24	30.53 ± 6.59	33.72 ± 5.304	0.32	31.6 ± 6.8	28.88 ± 7.1	0.21

#### Laboratory data during VA-ECMO support

Patients with neurological events had hypoalbuminaemia and higher blood glucose and serum creatinine levels compared to those without CNS events. The peak lactate level and lactate after 24 hours of ECMO support were significantly higher in those with CNS events.

Patients with ICH had significant thrombocytopenia and higher INR with more prolonged aPTT and PTT ratio than those with ischaemic stroke. Patients with ICH had higher serum bilirubin and serum lactate at 24 hours while lesser serum albumin than those with confirmed ischaemic strokes. The peak lactate and blood sugar levels were higher in the ischaemic group as compared to the ICH group (Table 4).

**Table 4:** Laboratory criteria during VA-ECMO support

Laboratory data	All patients	CNS events	No CNS events	*p* value	Ischaemic stroke	ICH	*p* value
Platelet count (10^9^/L)	101.43 ± 77.09	89.32 ± 65.81	117.31 ± 37.63	0.103	144.50 ± 169.86	55.63 ± 29.3	0.003
aPTT (seconds)	57.79 ± 16.26	57.39 ± 20.9	58.31 ± 6.62	0.8	47.20 ± 8.7	64.13 ± 14.5	0.001
PTT ratio	1.79 ± 0.54	1.68 ± 0.66	1.93 ± 0.25	0.06	1.5 ± 0.51	2.1 ± 0.23	0.03
INR	2.72 ± 2.15	3.37 ± 2.49	1.86 ± 0.35	0.06	1.6 ± 0.51	5.15 ± 2.21	0.001
Fibrinogen level (g/L)	3.12 ± 1.53	2.95 ± 1.59	3.34 ± 1.42	0.28	3.80 ± 1.6	2.13 ± 0.9	0.06
Peak lactate (mmol/L)	14.67 ± 4.37	15.68 ± 3.98	13.34 ± 4.57	0.033	16.50 ± 2.8	15 ± 4.9	0.04
Lactate at 24 hours	5.97 ± 5.13	7.11 ± 5.49	4.48 ± 4.24	0.031	6.40 ± 5.2	7.13 ± 5.7	0.01
Serum creatinine (μmol/L)	140.04 ± 69.45	131.45 ± 53.34	151.31 ± 85.91	0.04	131.6 ± 54.5	129.5 ± 48.9	0.7
Blood urea (mmol/L)	13.97 ± 8.03	14.18 ± 8.34	13.69 ± 7.74	0.81	16.10 ± 9.7	15.88 ± 8.7	0.3
Serum bilirubin (μmol/L)	172.60 ± 109.69	238.28 ± 198.63	138.48 ± 92.51	0.14	199.5 ± 83.1	341.8 ± 182.1	0.01
Highest blood glucose (mmol/L)	16.93 ± 3.41	18.03 ± 2.87	15.48 ± 3.57	0.003	18.80 ± 2.7	16.75 ± 4.1	0.01
Lowest blood glucose (mmol/L)	5.13 ± 1.61	4.84 ± 1.38	5.52 ± 1.83	0.10	5 ± 1.3	4.5 ± 1.2	0.18
Serum albumin (g/L)	32.40 ± 5.35	30.63 ± 5.59	34.72 ± 4.05	0.001	33.7 ± 5.3	26.75 ± 5.7	0.001

**Fig. 1:**
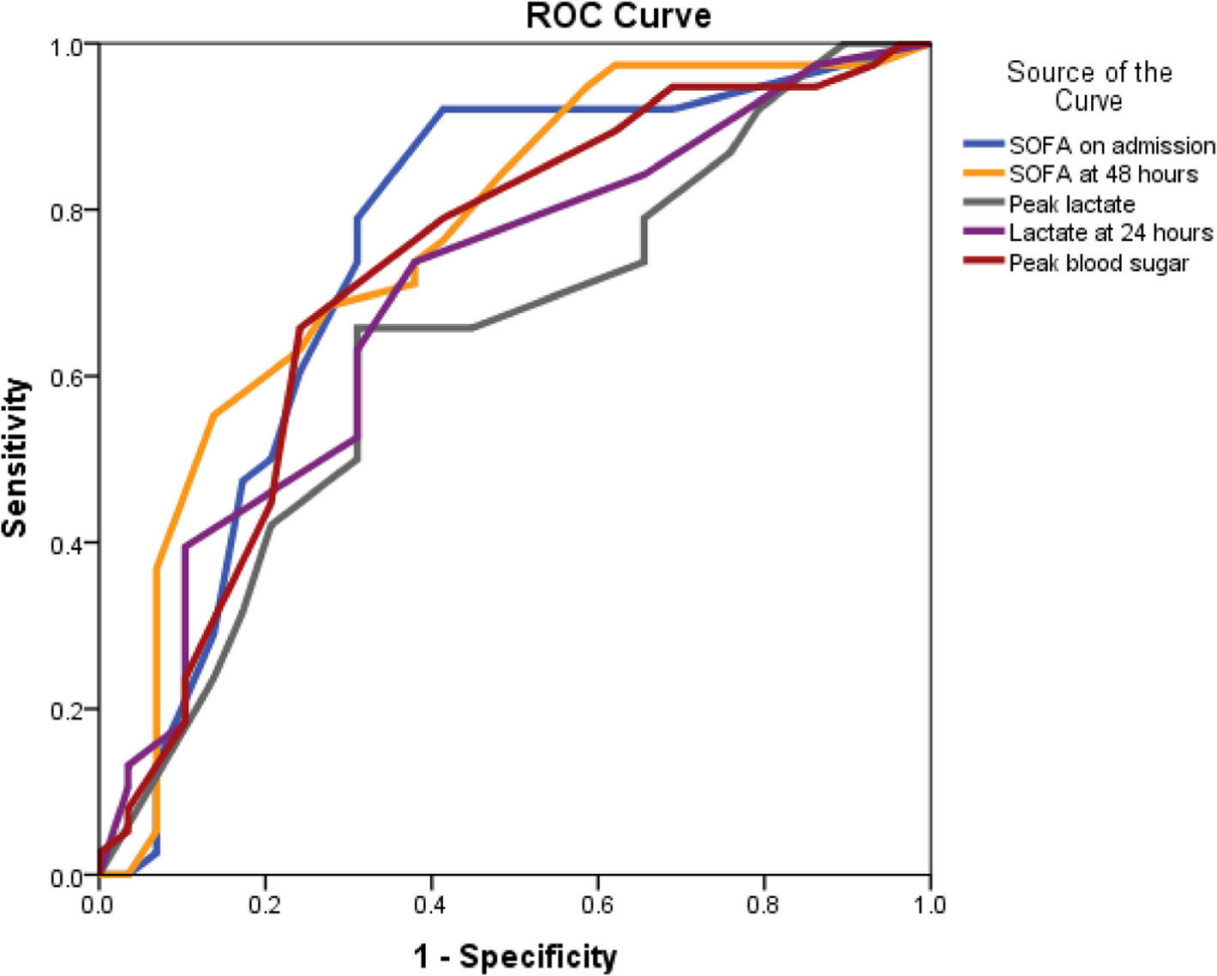
ROC curves of SOFA, lactate and blood sugar for predicting CNS complications

SOFA score over 10.5 measured upon ICU admission had a 92.1% sensitivity and 58.6% specificity for predicting CNS complications [area under the curve = 0.75, 95% confidence interval (CI) 0.62–0.88; *p* < 0.001 ] while SOFA over 18.5 measured after 48 hours of admission had a 55.3% sensitivity and 86.2% specificity for predicting CNS complications [area under the curve = 0.77, 95% CI 0.65–0.89; *p* < 0.001 ]. The peak lactate level over 15.5 mmol/L measured after VA-ECMO insertion had a 65.8% sensitivity and 69% specificity for predicting CNS complications [area under the curve = 0.64, 95% CI 0.51–0.78; *p* = 0.05 ] while lactate level over 3.5 mmol/L measured after 24 hours of VA-ECMO support had a 73.7% sensitivity and 62.1% specificity for predicting CNS complications [area under the curve = 0.71 , 95% CI 0.57–0.83; *p* = 0.006 ]. The peak blood glucose level over 17.5 measured after VA-ECMO insertion had a 65.8% sensitivity and 75.9% specificity for predicting CNS complications [area under the curve = 0.73, 95% CI 0.59–0.85; *p* = 0.002 ] (Fig. 1).

### Outcomes of patients with VA-ECMO

**Fig. 2:**
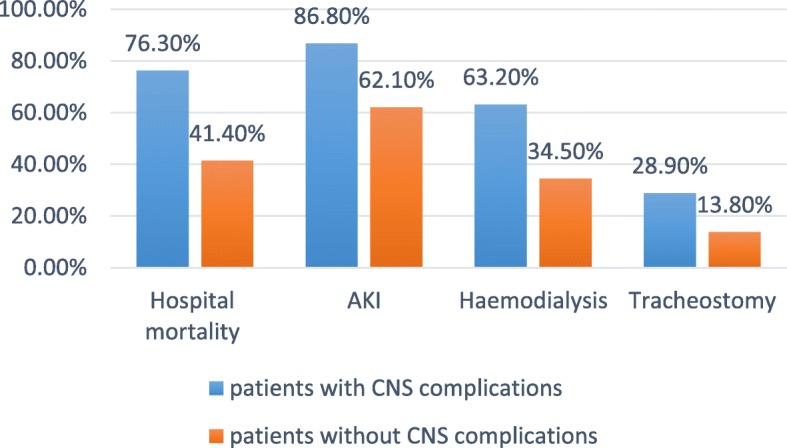
Hospital outcome of patients with and without CNS complications

Patients with acute neurological events had significant hospital mortality, more mechanical ventilation days, tracheostomy, AKI and haemodialysis compared with those without CNS events, but there were no significant differences between both groups regarding ECMO duration, ICU or post ICU stays nor 1 year mortality (Table 5, Fig. 2).

**Table 5:** Outcomes of VA-ECMO-treated patients

Characteristics	All patients	CNS events	No CNS events	*p* value	Ischaemic stroke	ICH	*p* value
ECMO duration (days)	9.91 ± 7.51	10.79 ± 7.28	8.76 ± 6.34	0.26	13.10 ± 8.6	10.88 ± 6.9	0.19
ICU stay (days)	47.4 ± 34.3	59.9 ± 42.4	23.7 ± 18.8	0.07	94.9 ± 57.6	33.7 ± 21.4	0.01
Mechanical ventilation (days)	45.4 ± 26.6	57.9 ± 34.6	16.17 ± 14.799	0.02	48.3 ± 26.2	28.1 ± 21.5	0.02
Cerebral oximetry (rSO2%)	45.76 ± 11.64	37.21 ± 7.07	56.97 ± 4.92	0.04	36.70 ± 5.2	34.25 ± 5.2	0.8
Tracheostomy	15 (22.4)	11 (28.9)	4 (13.8)	0.034	2 (50)	3 (37.5)	0.31
Acute kidney injury	51 (76.1)	33 (86.8)	18 (62.1)	0.023	8 (80)	7 (87.5)	0.63
Haemodialysis	34 (50.7)	24 (63.2)	10 (34.5)	0.027	6 (60)	6 (75)	0.42
Post ICU hospital stay (days)	16.6 ± 9.4	16.9 ± 7.0	15.9 ± 12.5	0.17	20.3 ± 7.9	8.13 ± 2.8	0.06
Hospital mortality	41 (61.2)	29 (76.3)	12 (41.4)	0.005	7 (70)	7 (87.5)	0.04
1 year mortality	0	0	0		0	0	
Tube feeding at home	4 (5.9)	4 (10.5)	0	0.006	2 (20)	1 (12.5)	0.15
Tracheostomy breathing at home	3 (4.5)	3 (7.9)	0	0.02	1 (10)	1 (12.5)	0.6
Chronic haemodialysis	1 (1.5)	1 (2.6)	0	0.32	0	1 (12.5)	0.18

As compared to those with ischaemic strokes, patients with ICH had higher in-hospital mortality, less ICU and post ICU stays and less mechanical ventilation duration. After hospital discharge, tube feeding and tracheostomy breathing were significantly frequent in the patients with CNS complications but without mortality difference during the 1 year follow-up (Table 5).

### Hospital outcomes according to VA-ECMO types

We compared between the studied patients regarding the type of VA-ECMO support. The patients supported with central VA-ECMO had significant hospital mortality without statistically significant morbidities as compared to those supported with peripheral VA-ECMO. The mean SOFA score of patients with central VA-ECMO was significantly higher at ICU admission and after 48 hours assessment as compared to patients with peripheral VA-ECMO. There were no significant differences between both groups regarding CPB or aortic cross-clamping times nor the time of ECMO initiation indicated by serum lactate level (Table 6).

**Table 6:** Comparison between central and peripheral VA-ECMO

Studied criteria	Central VA-ECMO (*n* = 35, 52.2%)	Peripheral VA-ECMO (*n* = 32, 47.8%)	*p* value
ECMO days	9.4 ± 7.2	10.5 ± 6.7	0.56
Ischaemic stroke (*n*, %)	5 (14.3%)	5 (15.6%)	0.5
ICH (*n*, %)	6 (17.1%)	2 (6.2%)	0.07
AKI (*n*, %)	30 (85.7%)	21 (65.6%)	0.051
Haemodialysis (*n*, %)	20 (57.1%)	14 (43.8%)	0.19
Serum lactate at ECMO initiation (mmol/L)	6.7 ± 2.4	5.8 ± 1.9	0.13
Peak lactate (mmol/L)	15.5 ± 4.6	13.7 ± 3.9	0.08
Lactate at 24 h (mmol/L)	6.8 ± 5.2	5.1 ± 4.2	0.18
CPB time (minutes)	240.2 ± 96.8	183.1 ± 87.3	0.16
Aortic cross clamping (minutes)	156.1 ± 56.2	122.4 ± 52.7	0.18
Initial SOFA	14.5 ± 3.4	11.6 ± 3.1	0.003
SOFA at 48 hours	16.2 ± 5.8	13 ± 4.6	0.02
Hospital mortality (*n*, %)	25 (71.4%)	16 (50%)	0.04

## Discussion

Our study revealed the high frequency of acute neurological events in patients with cardiogenic shock and supported with VA-ECMO (mostly postcardiotomy), and these CNS events were associated with significant morbidity and in-hospital mortality. Ischaemic stroke was diagnosed in 14.9% of patients while ICH was diagnosed in 11.9% of patients and was associated with higher mortality.

Brain injury is a well-known complication of ECMO support. Nasr et al. reviewed 23,951 patients from the Nationwide Inpatient Sample and found 4.1% ischaemic stroke and 3.6% intracranial bleeding [4]. Although that study included a large number of patients, they assessed both VV- and VA-ECMO together and did not examine the risk factors for neurological complications. Gray et al. studied 2000 ECMO-treated adult patients for acute heart failure and found that there was 8% ischaemic stroke and intracranial bleeding with a 43% survival rate [11]. That study combined ischaemic and haemorrhagic strokes and did not evaluate risk factors for the neurological injury. Our study revealed VA-ECMO-treated patients developed 14.9% ischaemic stroke and 11.9% ICH with 76.3% in-hospital mortality compared to patients without acute neurological events. Our study revealed a significant mortality difference between patients with neurological events and those without events, and the patients with ICH had a significantly higher mortality than those with ischaemic stokes.

Lorusso et al. evaluated 4522 ECMO-treated patients included in the registry of Extracorporeal Life Support Organization, detected 15.1% neurological complications occurred during VA-ECMO and identified pre-ECMO cardiac arrest, use of inotrope on ECMO and hypoglycaemia as risk factors for brain injury. The reported hospital mortality of patients with brain injury reached 89% and was significantly higher than those without brain injury 57% [5].

Recently, Le Guennec et al. studied retrospectively 878 VA-ECMO-treated patients and detected 5.3% ischaemic strokes and 2.8% intracranial bleeding and ICH rather than ischaemic stroke that was associated with higher mortality. Also, that study found that ischaemic strokes occurred after 1 week on ECMO support without specific risk factors and without increased mortality while ICH occurred earlier and was associated with female sex, central VA-ECMO, low platelet count and rapid CO2 change at ECMO start and high mortality [12].

In our work, there was no significant difference between onset of stroke or ICH and ECMO initiation, and the higher incidence of neurological manifestations in our study may be explained by the higher rates of cardiac surgeries in our studied patients. ECMO was not the only risk factor for neurological events in our patients; cardiac surgery, atrial fibrillation, intracardiac thrombi, haemodynamic instability before ECMO initiation, low cerebral blood flow due to advanced heart failure or other conditions may be also incriminated. Thus, it is very difficult to know the respective effect of the underlying disease(s) and ECMO itself on the development of neurological complications.

In our study, ICH was associated with low body mass index without sex differences, central more than peripheral ECMO, cardiothoracic surgeries, longer cardiopulmonary bypass and aortic cross-clamping times, thrombocytopenia and higher aPTT and INR while ischaemic strokes were associated with atrial fibrillation, diabetes mellitus, intracardiac thrombi and use of IABP.

Regarding analysis of the haemostasis and anticoagulation parameters of our patients, aPTT and PTT ratio were significantly higher at those with CNS events and those who developed ICH had significant thrombocytopenia during initiation of ECMO. Patients who developed ICH had significant thrombocytopenia and higher aPTT and INR compared to those with ischaemic strokes. Few studies have analysed the risk factors for ICH in VA-ECMO patients. Le Guennec et al. [12] did a multivariable analysis and found that at ECMO initiation, platelet count less than 100 (10^9^/L) was associated with ICH while platelet count > 350 (10^9^/L) was associated with ischaemic strokes and no relation between other haemostatic parameters or anticoagulation with brain injury. Sandersjöö et al. [13] reported ICH in 21% of 253 VV- and VA-ECMO-supported patients and that pre-ECMO antithrombotic treatment and low platelets on ECMO were associated with ICH. Kasirajan et al. [14] studied 78 VA-ECMO-treated patients, among whom 18.9% had ICH and the ICH was associated with female sex, renal failure and thrombocytopenia.

Regarding the analysis of the metabolic profile of our studied patients, the patients who developed CNS complications had significant hyperlactataemia (peak and after 24 h), hyperglycaemia and hypoalbuminaemia. Omar et al. evaluated risk factors in a combined VA- and VV-ECMO patients among whom 5.8% developed ischaemic strokes; their multivariable analysis detected pre-ECMO blood lactate > 10 mmol/L as being independently associated with strokes [15].

In our work, the peak lactate level over 15.5 mmol/L measured after VA-ECMO insertion had a 65.8% sensitivity and 69% specificity for predicting CNS complications (*p* = 0.05) while lactate level over 3.5 mmol/L measured after 24 h of VA-ECMO support had a 73.7% sensitivity and 62.1% specificity for predicting CNS complications (*p =* 0.006 ). The impact of hyperlactataemia on mortality has been documented in patients with cardiogenic shock and in those with cardiac arrest even if there is no cut-off value to be associated with worse outcomes or to guide haemodynamic and resuscitation management [16, 17].

We found significant hyperglycaemia in those who developed acute neurological manifestations especially in the ischaemic stroke while there was no significant difference regarding low blood sugar levels. We do not know if this finding was related to the cardiac surgeries and cardiopulmonary bypass or the extracorporeal circulation and inotropes used. Previous studies concluded that high glucose levels during and after CPB is an independent predictor of morbidity and mortality in both diabetic and non-diabetic patients [18]. Patients with persistently elevated glucose levels have increased post-operative mortality and risk of cerebrovascular strokes [19, 20].

In our study, we assessed our patients at admission and after 48 hours with SOFA score to assess clinical severity and degree of organ failure due to its simplicity and validity in reflecting progressive organ failure and increased mortality [21–23]. SOFA score at admission was higher in patients who developed acute brain injury than those who did not have clinical neurological events. Also, it was higher in patients with ICH than those who developed ischaemic strokes. SOFA score after 48 h of admission increased in those with CNS complications especially in those who had ICH.

Finally, acute neurological complications were frequent in patients supported with VA-ECMO and associated with significant morbidity and hospital mortality, but it was not confirmed if these CNS complications were related to ECMO itself as a supportive modality or related to underlying diseases and critical conditions of patients, the cardiac surgeries or the deteriorated metabolic and haemostatic parameters.

## Conclusion

Acute neurological events are frequent in patients supported with VA-ECMO and associated with significant morbidity and in-hospital mortality. As compared to ischaemic strokes, ICH is more frequent in younger patients with lesser BMI, central VA-ECMO after cardiothoracic surgeries, thrombocytopenia and coagulopathy. Our findings may have major implications for the care of patients requiring VA-ECMO.

## Study limitations

Our study was a single-centre retrospective study, the total number of included patients was small and there were some potentially confounding factors that need further larger studies.

## Data Availability

The data used in this study are available with the corresponding author upon request.

## Abbreviations

ACT: Activated clotting time
AKI: Acute kidney injury
AF: Atrial fibrillation
aPTT: Activated partial thromboplastin time
BMI: Body mass index
CKD: Chronic kidney disease
CPB: Cardiopulmonary bypass
DM: Diabetes mellitus
EF: Ejection fraction
GFR: Glomerular filtration rate
IABP: Intra-aortic balloon pump
ICH: Intracerebral haemorrhage
ICIS: Integrated Compliance Information System
INR: International normalized ratio
NIRS: Near-infra-red spectroscopy
SOFA: Sequential Organ Failure Assessment
VA-ECMO: Veno-arterial extracorporeal membrane oxygenation
VV-ECMO: Veno-venous extracorporeal membrane oxygenation

## References

[1] Muller G, Flecher E, Lebreton G (2016). The ENCOURAGE mortality risk score and analysis of long-term outcomes after VA-ECMO for acute myocardial infarction with cardiogenic shock. Intensive Care Med.

[2] Abrams D, Combes A, Brodie D (2014). What’s new in extracorporeal membrane oxygenation for cardiac failure and cardiac arrest in adults?. Intensive Care Med.

[3] Zangrillo A, Landoni G, Biondi-Zoccai G (2013). A meta-analysis of complications and mortality of extracorporeal membrane oxygenation. Crit Care Resusc.

[4] Nasr DM, Rabinstein AA (2015). Neurologic complications of extracorporeal membrane oxygenation. J Clin Neurol.

[5] Lorusso R, Barili F, Mauro MD (2016). In-hospital neurologic complications in adult patients undergoing venoarterial extracorporeal membrane oxygenation: results from the extracorporeal life support organization registry. Crit Care Med.

[6] Xie A, Lo P, Yan TD, Forrest P (2017). Neurologic complications of extracorporeal membrane oxygenation: a review. J Cardiothorac Vasc Anesth.

[7] Sutter R, Tisljar K, Marsch S (2018). Acute neurologic complications during extracorporeal membrane oxygenation: a systematic review. Crit Care Med.

[8] Lazar HL, McDonnell M, Chipkin SR (2009). The Society of Thoracic Surgeons practice guideline series: blood glucose management during adult cardiac surgery. Ann Thorac Surg.

[9] Ferrari M, Quaresima V (2012). A brief review on the history of human functional near-infrared spectroscopy (fNIRS) development and fields of application. Neuroimage.

[10] Cui X, Bray S, Reiss AL (2010). Functional near infrared spectroscopy (NIRS) signal improvement based on negative correlation between oxygenated and deoxygenated hemoglobin dynamics. Neuroimage.

[11] Gray BW, Haft JW, Hirsch JC, Annich GM, Hirschl RB, Bartlett RH (2015). Extracorporeal life support: experience with 2000 patients. ASAIO J.

[12] Le Guennec L, Cholet C, Huang F (2018). Ischemic and hemorrhagic brain injury during venoarterial-extracorporeal membrane Oxygenation. Ann Intensive Care.

[13] Fletcher Sandersjoo A, Bartek J, Thelin EP (2017). Predictors of intracranial hemorrhage in adult patients on extracorporeal membrane oxygenation: an observational cohort study. J Intensive Care.

[14] Kasirajan V, Smedira NG, McCarthy JF (1999). Risk factors for intracranial hemorrhage in adults on extracorporeal membrane oxygenation. Eur J Cardiothorac Surg.

[15] Omar HR, Mirsaeidi M, Shumac J (2016). Incidence and predictors of ischemic cerebrovascular stroke among patients on extracorporeal membrane oxygenation support. J Crit Care.

[16] Perez P, Kimmoun A, Blime V, Levy B (2014). Increasing mean arterial pressure in cardiogenic shock secondary to myocardial infarction: effects on hemodynamics and tissue oxygenation. Shock.

[17] Englehart MS, Schreiber MA (2006). Measurement of acid-base resuscitation endpoints: lactate, base deficit, bicarbonate or what?. Curr Opin Crit Care.

[18] Doenst T, Wijeysundera D, Karkouti K (2005). Hyperglycemia during cardiopulmonary bypass is an independent risk factor for mortality in patients undergoing cardiac surgery. J Thorac Cardiovasc Surg.

[19] Lazar HL (2006). Hyperglycemia during cardiac surgery. J Thorac Cardiovasc Surg.

[20] Parsons MW, Barber PA, Desmond PM (2002). Acute hyperglycemia adversely affects stroke outcome: a magnetic resonance imaging and spectroscopy study. Ann Neurol.

[21] Ferreira FL, Bota DP, Bross A, Melot C, Vincent JL (2001). Serial evaluation of the SOFA score to predict outcome in critically ill patients. JAMA.

[22] Vincent JL, Moreno R (2010). Clinical review: scoring systems in the critically ill. Crit Care.

[23] Argyriou G, Vrettou CS, Filippatos G (2015). Comparative evaluation of Acute Physiology and Chronic Health Evaluation II and Sequential Organ Failure Assessment scoring systems in patients admitted to the cardiac intensive care unit. J Crit Care.

